# Severe euglycemic diabetic ketoacidosis of multifactorial etiology in a type 2 diabetic patient treated with empagliflozin: case report and literature review

**DOI:** 10.1186/s12882-020-01930-6

**Published:** 2020-07-15

**Authors:** Erasmia Sampani, Pantelis Sarafidis, Chrysostomos Dimitriadis, Efstratios Kasimatis, Dimitra Daikidou, Konstantinos Bantis, Alexios Papanikolaou, Aikaterini Papagianni

**Affiliations:** 1 Department of Nephrology, Hippokration Hospital, Aristotle University of Thessaloniki, Konstantinoupoleos 49, GR54642, Thessaloniki, Greece; 2 2nd Department of Obstetrics and Gynecology, Hippokration Hospital, Aristotle University of Thessaloniki, Thessaloniki, Greece

**Keywords:** Type 2 diabetes mellitus, Euglycemic diabetic ketoacidosis, SGLT-2 inhibitors

## Abstract

**Background:**

Sodium-glucose co-transporter-2 (SGLT-2) inhibitors are a relatively novel class of oral medications for the treatment of Type 2 DM with a generally acceptable safety profile. However, these agents have been associated with rare events of a serious and potentially life-threatening complication named euglycemic diabetic ketoacidosis (euDKA). euDKA is not identical with the typical diabetic ketoacidosis, as it often presents with serious metabolic acidosis but only mild to moderate glucose and anion gap elevation.

**Case presentation:**

We report a case of a 51-year old female with Type 2 DM treated with an SGLT-2 inhibitor, developing severe metabolic acidosis with only mild blood glucose elevation after a routine surgery. A careful evaluation of involved factors led to the diagnosis of euDKA, followed by cautious application of simple therapeutic measures that resulted in complete restoration of acidosis and glycemic control in less than 48-h.

**Conclusions:**

Euglycemic ketoacidosis is a rare but rather serious complication of SGLT-2 inhibitors use, often with a multifactorial etiology. Its atypical presentation requires a high level of awareness by physicians as early recognition of this complication can quickly and safely restore acid-base balance.

## Background

Diabetic ketoacidosis (DKA) is a serious metabolic complication of diabetes mellitus (DM). In the United States only, more than 130,000 cases with a primary diagnosis of DKA were reported in 2006, two-thirds of which in patients with type 1 DM (T1DM) and one third in patients with type 2 DM (T2DM) [1]. According to the American Diabetes Association the diagnostic criteria for DKA include blood glucose levels > 250 mg/dl, arterial pH < 7.3, anion gap > 12 mEq/L, HCO_3_^**−**^ < 15 mEq/L and the presence of ketones in blood and urine [1]. Metabolic ketoacidosis in diabetic patients can less commonly occur with only mild to moderate glucose elevation, thus called euglycemic DKA (euDKA). EuDKA was first described as a discrete entity by Munro et al. in 1973 who reported a series of 211 patients with DKA, 37 of which had a glucose concentration at presentation less than 16.7 mmol/L (300 mg/dL); all of these originally described patients were individuals with T1DM [2]. Consequent and larger epidemiologic studies on euDKA during the 1980s and 1990s reported an incidence of euDKA between 1 and 3.2% of patients presenting with DKA, i.e. suggesting it was rather a rare condition [3].

Sodium-glucose cotransporter-2 (SGLT-2) inhibitors represent one of the latest approved classes of oral medications for the treatment of T2DM. They can be used alone or in combination with other drugs in these patients. Their mechanism of action involves lowering plasma glucose concentration by inhibiting the reabsorption of glucose in the proximal renal tubule [4]. In 2015, the US Food and Drug Administration (FDA) released an official warning concerning an increased risk of DKA with uncharacteristically mild to moderate glucose elevation, i.e. euDKA, in patients on these agents [5]. This was associated with a number of cases of euDKA reported in patients with T2DM, for whom this class of agents is indicated, but there were also rare reports of serious DKA in T1DM patients, where the drug was used off-label. Α review of the FDA Adverse Event Reporting System database identified 73 cases of ketoacidosis, from March 2013 to May 2015, in patients with T1DM or T2DM treated with SGLT-2 inhibitors [6]. In many of those cases ketoacidosis was not immediately recognized because of only mild glucose elevation, below this expected for typical DKA (euglycaemic ketoacidosis). In major outcome trials with SGLT-2 inhibitors in patients with T2DM (EMPA-REG, CANVAS), the incidence of DKA was rare, ranging between 0.1 and 0.6%, and it was not different between the active and placebo groups [7, 8]. However, in a recently published retrospective cohort study the incidence of diabetic ketoacidosis in patients treated with SGLT-2 inhibitors was double (hazard ratio, 2.1; 95% [CI], 1.5 to 2.9) compared to that in patients treated with DDP4-inhibitor [9].

The pathophysiologic mechanism that leads to this serious metabolic disorder has been previously described. Accumulation of ketoacids in DKA, typically results in high anion gap metabolic acidosis. Hyperchloremic metabolic acidosis complicating diabetic ketoacidosis during the recovery phase has been well documented; however, its contribution at presentation of euglycemic DKA has been only recently recognized [10]. Furthermore, typical DKA commonly occurs in the presence of additional conditions (i.e. trauma, surgery or infection) that per se may lead to other coexisting types of metabolic acidosis (e.g. lactic, hyperchloremic). Herein, we describe a patient with T2DM under SGLT-2 treatment who 5 days post-surgery presented with severe acidosis and only mildly elevated anion gap, mild hyperglycemia and ketonuria. A traditional diagnostic approach based on serum glucose levels and the anion gap in this patient could have seriously misled us from the correct diagnosis.

## Case presentation

Our Nephrology team was asked to provide consultation for a 51-year-old female, hospitalized in the Department of Obstetrics and Gynecology who had a 2-day history of weakness, tachypnea, anorexia, vomiting, and mild abdominal pain. Six days prior to the consultation she underwent an elective hysterectomy due to uterine fibroids. Her past medical history included T2DM and peptic ulcer. She was a former smoker. Her medications included a combination of metformin/vildagliptin 850/50 mg b.i.d., empagliflozin 25 mg o.d. and omeprazole 20 mg o.d. The patient remained fasting for the 1st and 2nd postoperative day and, after feeding was re-instituted on the 3rd day, she reported episodes of vomiting during the 4th and the 5th postoperative day. On day 5, she also began to feel increasingly weak and fatigued, while on day 6 she became febrile, and was started on intravenous antibiotics (piperacillin/tazobactam 4.5 g t.i.d.)

On clinical examination, the patient appeared generally fatigued. Temperature was 37.7 °C, blood pressure was 160/80 mmHg, and heart rate 105 beats/min. The patient was tachypnoic with a respiratory rate of 35 breaths/min. Her abdominal examination revealed a fresh surgical incision, and diffuse tenderness with no focal guarding or rebound. The rest of the physical examination was unremarkable. Her diuresis was excessive, with a urine flow rate up to 300 ml/hour. Due to postoperative fever, blood and urine cultures were withdrawn, and an abdominal ultrasound was performed revealing no pathological findings. In patient’s initial laboratory investigation the white cell count was 31.2 K/μL (N:81%, L:7%, M:11%) and serum glucose level was 121 mg/dl. Serum creatinine was 0.67 mg/dl and urea 27 mg/dl, while albumin and calcium values were within the normal range. Liver function tests, bilirubin, and lipase were normal (Table 1). Arterial blood gas revealed severe metabolic acidosis, with a pH of 7.05, [HCO_3_^−^]: 3 mmol/L, PCO_2_: 12 mmHg, anion gap: 16.9 mEq/L, lactate: 0.6 mmol/L, [Na^+^]: 133 mmol/L, [K^+^]: 3.8 mmol/L and [Cl^−^]: 113 mmol/L.

**Table 1:** Initial and follow up laboratory values

	Initial	3 h post-treatment	12 h post-treatment	24 h post-treatment	48 h post-treatment
pH	7.05	7.21	7.31	7.43	7.47
HCO_3_^−^ (mmol/L)	3	7	10.5	17	18
PCO_2_ (mmHg)	12	18	21.4	25.5	25
Anion Gap (mEq/L)	16.9	17.6	15	10.3	10
Chloride (mmol/L)	113	111	113	107	106
Na / K (mmol/L)	133 / 3.8	136 / 4.2	138 / 4.1	134 / 3.4	134 / 4.9
Hct (%)	37.7	35	32	29.5	29.5
Glucose (mg/dl)	121			121	
Urea / Creatinine (mg/dl)	27 / 0.67			17 / 0.59	
Total protein / Albumin (g/dl)	6.7 / 3.5			5.5 / 2.8	
Phosphate (mg/dl)	–			0.7	1.7
Urine Ketones (mg/dl)	≥160			80	
Urine Glucose (mg/dl)	≥1000			≥1000	
Urine pH	5			5	

The patient was transferred in the Department of Nephrology, where during a more detailed examination of her medical history, she reported that she had continued taking her antidiabetic medication by herself, although this information was not formally recorded in her medical file and charts. Furthermore, she remained fasting for 48 h postoperatively. As severe acidosis with only mildly increased glucose levels was revealed, euDKA due to SGLT-2 inhibitor was highly suspected and blood and urine samples were drawn for ketone examination. The urinalysis was remarkable with more than 160 mg/dl of ketones, as well as more than 1000 mg/dl of glucose. The profound glycosuria with only mildly elevated blood glucose was highly suggestive of treatment with an SGLT-2 inhibitor, which act by inhibiting glucose reabsorption and are the only antidiabetic agents increasing urine glucose concentration.

With the diagnosis of euDKA confirmed, as HCO_3_^**−**^ levels were extremely low and the anion gap only mildly elevated, 100 mmol of NaHCO_3_ were initially infused over 2 h. In parallel, intravenous fluids and insulin administration were started based on DKA protocol. Serum HCO_3_^**−**^ levels were only minimally increased at 3 h, but with the DKA causes being removed and on the basis of normal renal function they were further improved over time. Blood gases at 12 and 24 h post the initiation of treatment are presented in Table 1, suggesting that the pH was normalized in 24 h. As profound hypophosphatemia with serum phosphate of 0.7 mg/dl was noted 24 h after admission, phosphate infusion was also administered (20 mmol of sodium glycerophosphate over 16 h). The patient regained her appetite and was switched to a basal and pre-prandial insulin regimen. At that time, clinical signs of postsurgical cellulitis became apparent. At 48 h, the patient was transferred back to the Department of Obstetrics and Gynecology for continuation of treatment for post-surgical infection with proper antibiotics. The laboratory results at 48 h are presented in Table 1. The patient was discharged 5 days later (Day 12) at good shape with antidiabetic regimen consisting only of metformin/vildagliptin 850/50 mg b.i.d. At 3 months the patient visited out Nephrology Outpatient Clinic; she reported no symptoms, had a pH of 7.37, [HCO_3_^**−**^] 25 mmol/L and normal renal function and she was referred back to her general practitioner for further follow-up.

## Discussion and conclusions

DKA is a serious complication of diabetes; it is more commonly observed in T1DM patients, but it also occurs in patients with T2DM, especially under conditions of extreme physical stress such as trauma, surgery or infection [1]. EuDKA, i.e. DKA without severe glucose elevation was previously considered a rare subtype of DKA presenting solely in T1DM patients [2, 3]; however, the introduction of SGLT-2 inhibitors resulted in increased occurrence of this complication not only in T1DM receiving these agents off-label, but also in T2DM patients. The central factor involved in ketoacidosis is the relative lack of insulin combined with high levels of competitive hormones including glucagon, cortisol and catecholamines. In this metabolic setting, free fatty acids are released from adipocytes into the bloodstream and delivered to the liver where they are directed towards ketogenesis [11]. Ketone bodies constitute then the basic metabolic fuel and are consumed through oxidation in the brain, heart and the kidneys. In the initial stages, glomerular filtration of ketone bodies increases and some of them are excreted in urine as sodium salts. From an acid-base perspective, excreted ketone bodies are equivalent to bicarbonates and therefore this loss represents an indirect NaHCO_3_ loss resulting in hyperchloremic metabolic acidosis. Within 4–5 days, allowing for the completion of renal ammoniagenesis, the excretion of ketone anions occurs in parallel with the formation of ΝΗ_4_^+^, an action that may restore the bicarbonate buffer system [12]. Thus, a hyperchloremic acidosis which may be present at the initial stages can turn to a high anion gap acidosis.

Of note, previous studies pointed out that in ketoacidosis ammoniagenesis is not fully sufficient for an optimal renal response to the ketoacid load. Ammoniagenesis implies glutamine oxidation and proximal tubular cells preferentially use ketones and free fatty acids, if available, for their energy needs to reabsorb sodium. Energy needs are anyway reduced in the case of SGLT-2 inhibitors because less sodium is reabsorbed in the proximal tubule [13]. The preference in ketones and free fatty acids as fuel probably serves to minimize protein breakdown during prolonged starvation. Patients with DKA often present with hypovolemia, due to osmotic diuresis and reduced glomerular filtration rate (GFR), leading to blunted renal response to acidosis and diminished removal of ketoacids via oxidation, resulting in marked ketonemia.

SGLT-2 inhibitors are novel and promising therapeutic treatments for patients with diabetes as they improve glucose homeostasis and weight loss while having a cardioprotective and renoprotective profile [4]. SGLT-2 is a sodium-glucose co- transporter located in the apical surface of renal proximal tubular cells. SGLT-2 expression is enhanced in T2DM and as it reabsorbs equimolar amounts of glucose and sodium, its inhibition promotes distal delivery of sodium and chloride along with glucosuria and loss of calories. SGLT-2 inhibitors are the only anti-diabetic drugs to increase glucose excretion; therefore, their use prevents high glucose levels in T2DM patients while plasma insulin and insulin to glucagon ratio remain low. As discussed above, the relative lack of insulin is central in ketoacidosis through stimulation of free fatty acid production from the liver and ketone body increase [11]. Furthermore, insulin intervenes with carnitine palmitoyltransferase–I (CPT-I), an enzyme promoting fatty acid entry into mitochondria. Low levels of insulin result in decreased production of malonyl-CoA, a potent inhibitor of CPT-I; this also increases β-oxidation rate and ketoacids production [14]. Ferranini et al. reported that in T2DM patients, fasting β-hydroxybutyrate (β-HB) levels doubled after empagliflozin administration [15]. The observed rise in β-HB was not due to reduced renal clearance but to overproduction, as both the clearance rate and fractional excretion of β-HB were increased [16].

Furthermore, SGLT-2 inhibition may increase glucagon levels through multiple mechanisms [17]. These include a direct effect on SGLT-2 proteins expressed in glucagon-secreting alpha pancreatic cells [18], or indirectly, i.e. acting on brain SGLT-2 receptors and promoting a cathecholamine increase, which in turn would stimulate glucagon secretion [19]. Thus, SGLT-2 inhibitor treatment may not only decrease insulin levels but also increase glucagon, and catecholamines. It was also suggested that the combination of insulinopenia and dehydration represent a two-hit model to provoke euDKA secondary to SGLT-2 inhibitors treatment [19]. Volume depletion is associated with further increases in both plasma cortisol and catecholamine and, although is considered temporal at initiation of treatment, it could reappear in case there is no free access to water and food.

The present case meets the criteria for the two-hit model proposed, as she was both insulinopenic due to starvation and volume depleted. There was a restriction in carbohydrate availability as the patient remained fasting for 48 h postoperatively and later developed nausea and vomiting, under conditions of well-known precipitants such as surgical stress and intercurrent postoperative infection. Regarding volume depletion, both oral and standard parenteral hydrations were inadequate to replace urine losses as a diuresis rate of 300 ml/h was recorded. This is indirectly supported by the fact that after volume restoration there was a marked decrease in hematocrit and serum proteins (Table 1). euDKA is a serious but rare complication of SGLT-2 inhibitors and, at least in T2DM patients, it is triggered by well-defined predisposing risk factors. For elective major surgical procedures, it is recommended to withhold SGLT-2 inhibitors at least 3 days prior surgery [20, 21]. A similar strategy to administer insulin instead of SGLT-2 inhibitors could prevent euDKA in the presence of other predisposing risk factors for DKA. A high level of awareness, including simple diagnostic measures, such as blood gas and basic biochemical and electrolyte panel, would ensure the diagnosis of DKA even with atypical features such as euglycemia and a near-normal anion gap. This is particularly relevant for patients in which preventive pre-surgery measures are incomplete, or in cases of acute surgery [20, 21].

Guidelines for the treatment of euDKA are not available. Relevant recommendations for the treatment of DKA suggest volume restoration with isotonic fluid of NaCl 0,9%, ensuring that serum K is > 3.3 mmol/l before initiation of insulin therapy, while bicarbonate infusion should only be considered in cases of life-threatening acidosis (PH < 6.9) [1]. However, recognition of the pathophysiologic mechanisms involved, enables successful treatment with simple measures. In our case in particular, infusion of a minimum amount of bicarbonate, along with proper insulin administration was sufficient to restore the HCO_3_^−^ deficit and to raise the pH into safe levels. Resolution of vomiting and proper feeding along with insulin administration and SGLT-2 inhibitor withdrawal completely reversed the abnormal metabolic process. In less than 24 h the patient had restored normal blood pH and was switched to subcutaneous insulin regiment.

Overall, euDKA associated with SGLT-2 inhibitor use in T2DM is a serious side-effect, often with a multifactorial etiology. Increased awareness and recognition of involved factors can safely restore normal acid-base balance with simple measures. Given the undisputed cardiovascular and renal benefits of SGLT-2 inhibitors and their awaited increased use, physicians need to familiarize themselves with euDKA and its treatment in order to readily manage this complication for the benefit of our patients.

## Data Availability

This is a report of a clinical case; all information relevant to the clinical course of the patient remain encrypted at our hospital’s archive and is not available outside our hospital.

## Abbreviations

DKA: Diabetic ketoacidosis
DM: Diabetes mellitus
T1DM: Type 1 diabetes mellitus
T2DM: Type 2 diabetes mellitus
SGLT-2: Sodium-glucose cotransporter-2
FDA: Food and drug administration
euDKA: euglycemic diabetic ketoacidosis
GFR: Glomerular filtration rate
β-HB: β-hydroxybutyrate
